# Perceived acceptability and preferences for low-intensity early activity interventions of older hospitalized medical patients exposed to bed rest: a cross sectional study

**DOI:** 10.1186/s12877-018-0722-6

**Published:** 2018-02-20

**Authors:** Mary T. Fox, Souraya Sidani, Dina Brooks, Hugh McCague

**Affiliations:** 1 York University Centre for Aging Research and Education, Faculty of Health, York University, HNES suite 343, 4700 Keele Street, Toronto, ON M3J 1P3 Canada; 2 Faculty of Community Services, Ryerson University, 350 Victoria Street, Suite YNG 316, Toronto, ON M5B 2K3 Canada; 3 Department of Physical Therapy, University of Toronto, 160-500 University Avenue, Toronto, ON M5G 1V7 Canada; 4 Institute for Social Research, DB 5064, York University, 4700 Keele Street, Toronto, ON M3J 1P3 Canada

**Keywords:** Early activity, Intervention acceptability, Intervention preference, Hospital, Medical patients, Older adults

## Abstract

**Background:**

Hospitalized older patients spend most of their time in bed, putting them at risk of experiencing orthostatic intolerance. Returning persons to their usual upright activity level is the most effective way to prevent orthostatic intolerance but some older patients have limited activity tolerance, supporting the need for low-intensity activity interventions. Consistent with current emphasis on patient engagement in intervention design and evaluation, this study explored older hospitalized patients’ perceived acceptability of, and preference for, two low-intensity early activity interventions (bed-to-sitting and sitting-to-walking), and characteristics (gender, illness severity, comorbidity, illnesses and medications with orthostatic effects, and baseline functional capacity) associated with perceived acceptability and preference.

**Methods:**

A convenience sample was recruited from in-patient medical units of two hospitals in Ontario, Canada and included 60 cognitively intact adults aged 65+ who were admitted for a medical condition within the past 72 h, spent ≥ 24 consecutive hours on a stretcher or in bed, presented with ≥ 2 chronic diseases, understood English, and were able to ambulate before admission. A cross-sectional observational design was used. Participants were presented written and oral descriptions and a 2-min video of each intervention. The sequence of the interventions’ presention was randomized. Following the presentation, a research nurse administered measures of perceived acceptability and preference, and collected health and demographic data. Perceived acceptability and preference for the interventions were measured using the Treatment Acceptability and Preferences Scale. Illness severity was measured using the Modified Early Warning Score. Comorbidity was assessed with the Age Adjusted Charlson Comorbidity Scale and the Cumulative Illness Rating Scale – for Geriatrics. Baseline functional capacity was measured using the Duke Activity Status Index.

**Results:**

Participants’ perceived acceptability of both interventions clustered above the scale midpoint. Most preferred the sitting-to-walking intervention (*n* = 26; 43.3%). While none of the patient characteristics were associated with intervention acceptability, illness severity (odds ratio = 1.9, *p* = 0.04) and medications with orthostatic effects (odds ratio = 9.9, *p* = 0.03) were significantly associated with intervention preference.

**Conclusions:**

The interventions examined in this study were found to be acceptable to older adults, supporting future research examining their feasibility and effectiveness.

## Background

Since the 1950s, getting up out of bed, as a minimum practice, has been part of recommended daily hospital care [1]. Nevertheless, on admission to hospital, older patients (aged 65+) spend most of their time on stretchers in emergency departments or in beds on in-patient units [2, 3]. Studies have found older patients to spend between 71% and 83% of their time in hospital lying down [3–5], with up to 40% remaining in bed for at least 24 h [6–8]. This exposure has been identified as the starting point in a trajectory to bed rest dependency in those with multiple chronic conditions [9]. Bed rest dependency is characterized by a compulsion to return to bed soon after getting up, self-limited upright activity, and/or refusal to get up [9].

Acutely-ill older patients with multiple chronic conditions are particularly vulnerable to bed rest dependency because they tend to use rest as a strategy for managing symptoms, such as fatigue, associated with chronic illnesses [9]. Many (25% to 34%) older patients believe walking and other exercises are inappropriate during hospitalization [10, 11] and that they need to stay in bed during illness and focus on recuperating, not on walking [10]. Others view the hospital as a dangerous place and prefer to remain inactive [10, 11]. Just a few days of bed rest can lead to rapid functional decline and related complications in this population because their physiologic reserves are diminished [6, 12]. Bed rest has been associated with functional decline, institutionalization, and mortality in hospitalized older patients even after controlling for comorbidity and illness severity [6]. Consequently, preventing bed rest dependency, early in its trajectory, is critical.

Informed by the Medical Research Council’s framework for designing and evaluating interventions [13], we critically analyzed theoretical and empirical evidence to generate a comprehensive understanding of bed rest dependency. This conceptualization guided us in developing two interventions to promote activity during the early phase of hospitalization for older patients with multiple chronic conditions. We developed two distinct interventions because, based on prior research [10, 11], we anticipated that patients’ perspectives would vary on their perceived need for rest and the appropriateness of different types and levels of activity during hospitalization for acute illnesses.

In this paper, we describe our conceptualization of bed rest dependency, the two low-intensity early activity interventions, and, in keeping with the Medical Research Council’s framework’s emphasis on engaging patients in intervention design, we describe older hospitalized patients’ perceived acceptability of, and preference for, those interventions. Perceived acceptability refers to the desirability of interventions to potential users; it reflects the extent to which patients anticipate the interventions to be appropriate and effective in addressing bed rest dependency and related health problems, with minimal side effects or risks, and ease of use in hospital [14]. The Medical Research Council’s framework acknowledges that intervention evaluations are frequently challenged by problems around acceptability that limit feasibility [15]. Assessing perceived acceptability and preferences is increasingly acknowledged as essential in the design of healthcare interventions, prior to determining their effectiveness [15, 16]. Interventions perceived as acceptable and preferred are more likely to be used than those that are not. Consequently, prior to testing the effectiveness of the interventions, it is important to better understand patients’ perspectives on them.

### Conceptualization of bed rest dependency

Adequate functioning of the cardiovascular system during physical activities such as walking is highly dependent on exposure to gravitational stress along the longitudinal axis of the body that normally accompanies an upright sitting or standing position [17]. During bed rest, the reclined position of a stretcher or bed furnishes a reduced gravitational environment along the longitudinal axis of the body to which the cardiovascular system quickly adapts (within two to four days) [17]. However, once bed rest has ended, the cardiovascular system has difficulty reversing this adaptation. Its compensatory mechanisms fail to adequately increase cardiac output and due to insufficient delivery of oxygen and nutrients to the tissues, the person experiences orthostatic intolerance [18]. Defined as reduced ability of the cardiovascular system to function effectively against gravitational stress, orthostatic intolerance is the hallmark of bed rest deconditioning and the most prominent determinant of self-limited upright activity after bed rest [19]. Orthostatic intolerance is manifested by symptoms of dizziness, tiredness, and weakness upon return to upright posture [20–22]. The severity of orthostatic intolerance is proportional to the duration of bed rest [23] and its recovery takes up to 3 times the duration of bed rest [24]. Symptoms (e.g. weakness, fatigue, dizziness) and fear of falling because of the symptoms are the reasons most frequently identified by patients and clinicians for older patients’ bed rest and reluctance to get up during acute care hospitalization [8, 11]. Avoiding the negative effects of bed rest, however, has been identified by older patients as a crucial factor in their motivation to engage in activity during hospitalization [11].

Based on this evidence, we developed a conceptualization of bed rest dependency which proposes that the amount of time spent lying down: (a) intensifies its negative effects on the cardiovascular system, and (b) perpetuates a cycle of bed rest dependency and functional decline, both of which in turn reinforce further bed rest dependency. Since the orthostatic effects are experienced when upright and relieved when recumbent, the dependency evolves from the person’s belief that resting in bed is the best way to manage the increasing symptoms and accompanying fears (e.g. falling and exacerbating health problems because of weakness and dizziness). This conceptualization supports the need to intervene early in the cycle by minimizing recumbency, promoting low-intensity activity facilitating vascular return, engaging patients in discussions about the negative effects of bed rest, preventing misconceptions about bed rest and motivating activity engagement.

### Empirical basis for interventions to prevent bed rest dependency on medical units

Evidence derived from controlled trials in laboratory settings with young to middle-aged adults has established that early activity, in the form of returning persons to their usual upright activity level, is the most efficient and effective non-invasive way to prevent and rehabilitate orthostatic intolerance [24–29]. Early activity in the hospital setting delivered during the acute phase of an illness has been minimally evaluated as a single component intervention and its effect on orthostatic intolerance has not been examined in older patients with multiple chronic conditions admitted to medical units. Rather, early activity has often been embedded in a multi-component program implemented in Acute Care for Elders (ACE) units, which has demonstrated effectiveness in reducing older patients’ functional decline and related outcomes [30].

Although studies that evaluated the ACE program did not isolate and test the unique contribution of early activity to outcome achievement, some studies which attempted to do so demonstrated poor feasibility because of patient refusal to participate [31, 32]. Brown reported a drop-out rate of 90% in an early activity program, consisting of progressive ambulation and transfer and resistance training, because it was viewed by older medical patients as too physically demanding [31]. Low-intensity early activity, consisting of walking, demonstrated better feasibility in older medical patients, but the studies included few women (≤ 3%), reported only moderate adherence because of refusals to walk and poor tolerance in some patients, and did not examine patients’ perceptions of the interventions [33, 34]. Older patients’ perceptions of early activity may vary with their gender [35, 36], illness severity [37], comorbidities [38], medications [39] and pre-hospital baseline functional capacity [4], but studies have not explored the influence of these characteristics on hospitalized older patients’ perceptions of early activity.

Taken together, prior research suggests the need to develop early activity interventions that can be used by patients who remain sedentary during hospitalization and are thus at risk of experiencing orthostatic intolerance when they resume ambulation. Yet, little is known about hospitalized older patients’ perceived acceptability of, and preferences for, early activity, which is essential to designing interventions to which patients will adhere [16, 40]. Consequently, consistent with our conceptualization and review of empirical evidence, we developed two low-intensity early activity interventions aimed at preventing orthostatic intolerance as well as consequent bed rest dependency and functional decline in older patients with multiple chronic conditions. The interventions are: 1) bed-to-sitting, and 2) sitting-to-walking (Table 1). The content of the interventions (i.e. the activities) was derived from studies that were predominantly conducted in laboratory settings. The bed-to-sitting-intervention was developed based on evidence suggesting that the orthostatic effects of bed rest can be mitigated through regular exposure to orthostatic stress (e.g. sitting upright with legs dependent) [28] and activation of the muscle venous pump of the legs (e.g. elevating legs, pointing toes) [41, 42]. The sitting-to-walking intervention was designed based on the same evidence, but its activities were more intensive (e.g. walking) with a stronger dose (e.g. being upright for at least 3.5 h in a 24-h period) based on prior evidence indicating that the orthostatic effects of bed rest are mitigated by conditions simulating upright posture for this duration [21, 26]. Both interventions included sitting at the bedside for up to 10 min prior to ambulating, consistent with time frames for the development of the symptoms of orthostatic intolerance [43].

**Table 1:** Descriptions of low-intensity early activity interventions

	Intervention	
	Bed-to-sitting	Sitting-to-walking
Goals	- To facilitate vascular return. - To reduce dizziness, tiredness and unsteadiness on feet upon ambulation following bed rest. - To prevent misconceptions about bed rest. - To ultimately prevent bed rest dependency and functional decline.	- To facilitate vascular return. - To reduce dizziness, tiredness and unsteadiness on feet upon ambulation following bed rest. - To prevent misconceptions about bed rest. - To ultimately prevent bed rest dependency and functional decline. - To train the body to get used to being up again.
Activities	- Point and flex toes while lying supine. - Slide feet back and forth toward buttocks, one at a time. - Lift buttocks up and down, as high as tolerated. - Slide legs, one at a time, out to side and back again. - Lift feet up and down, one at a time. - Sit on edge of bed with feet against floor for up to 10 min. - While sitting, lift heels up and down. - While sitting, lift knees up and down. - Return to lying in bed.	- Sit on edge of bed with feet against floor for up to 10 min. - While sitting, lift heels up and down. - While sitting, lift knees up and down. - Stand and sit in chair with feet touching the floor, and/or walk as tolerated.
Mode	- In bed and sitting on edge of bed	- Sitting at side of bed or chair and walking in room or hallway.
Dose	- Within 48 h of admission. - Each individual exercise is performed 10 times at least twice a day.	- Within 48 h of admission. - At least twice a day. - Number and length of sessions gradually increased until sitting in a chair with feet touching the floor and/or walking ≥ 3.5 h/ day as tolerated.

### Study aims

The purpose of this study was to examine the acceptability of, and preferences for, the bed-to-sitting and sitting-to-walking interventions, as perceived by older patients with multiple chronic conditions admitted to medical units in acute care hospitals. Specifically, we aimed to explore hospitalized older patients’: 1) perceived acceptability of, and preference for, the two interventions, 2) characteristics (gender, illness severity, comorbidity, illnesses and medications with orthostatic effects, baseline functional capacity) associated with acceptability and, 3) characteristics associated with preference.

## Methods

### Design

A cross-sectional observational design was used to address the study aims. Eligible, consenting patients attended a data collection session, held in their room, and led by a Master’s prepared research nurse with experience working in internal medicine. The nurse explained that people who stay in bed for a few days may feel dizzy, tired and unsteady on their feet and may have trouble walking when they first get up. She explained that the two interventions were developed based on previous research to help reduce these problems, and played a 2-min video of each intervention. Following each video, the nurse provided patients with a written handout describing each intervention and verbally explained (using a standard script) what the interventions entailed, clarifying the activities patients were to perform (e.g. lift your heels up and down), dose (e.g. 10 times), anticipated benefits (e.g. reducing dizziness), and possible side effects (e.g. tiredness if a lot of time had been spent lying down before initiating the interventions). The nurse explained when patients should expect any side effects to abate, based on evidence indicating that the effects of bed rest on orthostatic intolerance can last up to three times the duration of bed rest [24], and then administered the measures of perceived acceptability and preference for the interventions. The sequence of the presentation of the interventions was randomized using a computer-generated randomization scheme to control for possible order effects. A $10 incentive was provided in the form of a coffee shop gift card.

### Setting and sample

Participants were recruited from the in-patient general internal medicine units of two hospitals located in the Greater Toronto Area of Ontario, Canada in 2011. The units provide acute care services to patients with complex medical conditions and were comparable on key patient characteristics (e.g. age, chronic illnesses, reason for admission).

The convenience sample included adults aged 65 years and older, who had been admitted for the acute management of a medical condition within the past 72 h. Patients were eligible if they had spent ≥ 24 consecutive hours on a stretcher or in bed, presented with 2 or more chronic diseases identified on the International Classification of Disease-10, understood English, were ambulatory 2 weeks prior to admission, had a Mini Mental Status Exam (MMSE) score ≥ 24 [44] and a non-positive Confusion Assessment Method (CAM) [45]. Patients with a current medical order for bed rest and/or complete lower limb paralysis were excluded because the activity interventions were not applicable to their conditions. Patients admitted for palliation were also excluded on the basis that they may have chosen to remain in bed for comfort reasons.

The sample size of 60 was adequate to detect statistically significant associations of moderate magnitude based on the principle of 10 cases per predictor for 6 predictors [46] representing the patient characteristics.

### Measures

Screening measures included the MMSE and CAM; both have established psychometric properties [44, 45]. Perceived acceptability and preference for the two low-intensity early activity interventions were measured using the Treatment Acceptability and Preferences (TAP) scale, which demonstrated internal consistency reliability (alpha > .80) and factorial validity in prior research [47]. The TAP consisted of two sections. The first provided a written description of each intervention followed by a set of items to rate its perceived acceptability relative to its anticipated appropriateness, effectiveness, side effects/risks, and ease of use. A five-point scale that ranged from 0 *(not at all*) to 4 (*very much*) was used in the rating. The total score was derived by taking the mean of the items’ scores. The second section contained two questions to assess intervention preference: a dichotomous question asking if participants preferred one intervention over the other and, if so, a question asking them to indicate that preference.

Illness severity was measured using the Modified Early Warning Score (MEWS), a scale that was designed specifically for patients admitted under medical service [48]. The scale demonstrated predictive validity; scores ≥ 3 were found to predict admission to the intensive care units in middle aged patients [49] and death in older patients [50]. The MEWS requires extracting routinely collected data on five parameters (e.g. heart rate) from the medical chart, assigning a score to the value of the parameter, and then summing the scores to generate a total scale score. Total scale scores can range from 0 to 14 (higher scores reflect greater illness severity), and a score of 4 is a trigger for urgent medical review [51]. We extracted the first three hospital admission assessments and, where none of the individual MEWS parameters (e.g. pulse) were missing, we calculated total scale scores for the first three measurement occasions and used the mean of these scores.

Because of the lack of consensus on how to measure comorbidity [52], two established scales were used: the Age Adjusted Charlson Comorbidity Index (ACCI) [53, 54] and the Cumulative Illness Rating Scale – for Geriatrics (CIRS-G) [55] by medical chart review. The ACCI demonstrated predictive ability of 90-day and 5-year mortality [56, 57]. The ACCI requires users to indicate the presence of 19 conditions (e.g. congestive heart failure) which have pre-defined weights of 1 (e.g. congestive heart failure), 2 (e.g. hemiplegia), 3 (e.g. moderate or severe liver disease) or 6 (e.g. metastatic solid tumor) [53, 54]. The total scale score was derived by summing the weights for the conditions that are present, which can range from 0 to 37, [53] and then adding one point for each decade of life from age 50 on [54]; higher scores indicate higher comorbidity.

The CIRS-G demonstrated test-retest reliability in older medical patients and construct validity manifested by high correlations with other comorbidity scales [58] and by significantly predicting hospital readmission and one- and 5-year mortality rates [59]. The CIRS-G requires users to rate illnesses in 14 organ systems using a five-point severity scale ranging from 0 (*no impairment*) to 4 (*extremely severe impairment*). The total scale score, which can range from 0 to 56, was derived by summing the ratings in the 14 organ systems; higher scores indicate higher comorbidity.

Baseline functional capacity was measured using the Duke Activity Status Index (DASI), an established self-report scale which was designed for use in adults with chronic disease and has shown good psychometric properties [60]. The time referent “at this time” was revised to “two weeks before your current hospital admission” to establish baseline. DASI uses a weighted sum in which a weight based on the known metabolic cost of each activity in MET (metabolic equivalent) units is individually applied to *yes* responses to questions asking about the ability to perform 12 physical activities (e.g. climbing a flight of stairs); the weighted values are then summed to generate scores ranging from 0 to 58.20, with higher scores indicating higher functional capacity [60].

Information on gender as well as illnesses and medications with orthostatic effects, were extracted from the medical chart using a data collection tool developed by our team and used previously. Demographic and other health related data were collected to examine patient characteristics associated with perceived acceptability and preference as well as to describe the sample. Age, reason for admission, mobility devices used, and amount of time spent in the emergency department were extracted from the medical chart. The latter was calculated by subtracting the time of admission to the unit from the time of admission to the emergency department. Information on highest level of education and living situation were obtained from the patients using standard questions.

### Data analysis

Descriptive statistics were used to characterize the sample’s characteristics, as well as their perceived acceptability and preference for the two interventions (aim 1). Prior to conducting the regression analyses exploring the factors associated with perceived acceptability and preferences (aims 2 and 3), the data were examined for departures from normality and violations of the assumptions underlying regression. In addition, collinearity diagnostics were performed to determine if the independent variables were strongly correlated with each other, evidenced by tolerance values less than 0.1 and variance inflation factors (VIF = 1/tolerance) greater than 10 [61]. The forced entry method was used in all regression analyses.

Linear regression using ordinary least squares was employed to explore patient characteristics (gender, illness severity, comorbidity, illnesses and medications with orthostatic effects, and baseline functional capacity) associated with perceived acceptability (aim 2); these were performed separately for the two comorbidity measures (ACCI and CIRS-G) to reduce the potential for multicollinearity. Statistically significant predictors had beta weights with associated *P*-values ≤0.05. The forced entry method was used in all regression analyses.

Ordinal logistic regression analysis was used to explore the association of the same patient characteristics with preference (aim 3); these were also performed separately for the two comorbidity measures. Preference was defined by three ordinal groups represented by preference for the bed-to-sitting intervention (scored − 1), no preference (scored 0), and preference for the sitting-to-walking intervention (scored + 1). Statistically significant predictors had odds ratio (OR) > 1 and associated *P*-values ≤0.05.

## Results

### Participant characteristics and average standing on perceived early activity acceptability and preference

In total, 100 patients agreed to meet with the study nurse but 33 declined to participate in the study. Of the 67 who consented, 6 were ineligible because they did not have the minimum score on the pre-screening measures, and one withdrew after consenting. As shown in Table 2, the average patient was female, aged 79 years old, lived alone and used a cane to walk.

**Table 2:** Characteristics of 60 study participants

Variable	
Age—mean ± SD	79 ± 8.2
Gender—n (%)	
Women	32 (53)
Men	28 (47)
Living Situation before admission—n (%)	
Lives alone in private home	23 (38.3)
Lives with spouse or another adult in private home	28 (46.7)
Lives in assisted housing	9 (15.0)
Highest level of education—n (%)	
Elementary school	14 (23.3)
High school	26 (43.3)
Vocational or trade school	7 (11.7)
University (bachelors and masters)	13 (21.6)
Hours spent in emergency department—median (range)	23.8 (5.5–123)
Reason for hospital admission^a^—n (%)	
Pneumonia and chronic obstructive pulmonary diseases	15 (25)
Other diseases (e.g. ketoacidosis, cancer, fever, hypoglycemia, renal failure and sepsis)	13 (21.7)
Congestive heart failure, acute coronary syndrome and other heart diseases	10 (16.7)
Diarrhea and gastroenteritis	7(11.7)
Syncope, transient ischemic attack, and vertigo	7 (11.7)
Failure to cope	4 (6.7)
Gastrointestinal bleed	3(5.0)
Cellulitis and other skin infections	3(5.0)
Anemia and pancytopenia	3(5.0)
Illnesses with orthostatic effects—n (%)	
Yes	52 (86.7)
No	6 (13.3)
Medications with orthostatic effects—n (%)	
Yes	53 (88.3)
No	7 (11.7)
Mobility devices—n (%)	
Cane	27 (45)
None	23 (38.4)
Walker	10 (16.7)
MEWS—mean ± SD	2.0 ± 1.06
MEWS—median (range)	2 (0.50–5.0)
DASI—median (range)	7.2 (0–58.20)
CIRS-G—mean total scale score ± SD	15.77 ± 4.69
Number of CIRS-G organ systems rated at level 4—n (%)	
0	5 (8.3)
1	30 (50)
2	22 (37)
3	3 (5)
ACCI—mean total scale score ± SD	5.88 ± 1.91

*ACCI* Age Adjusted Charlson Comorbidity Index, *CIRS-G* Cumulative Illness Rating Scale – for Geriatrics, *DASI* Duke Activity Status Index, *MEWS* Modified Early Warning Score, *n* number, *SD* standard deviation

^a^Numbers do not total 60 because some patients had more than one reason for admission reported. All other diseases had less than 2 cases in each disease category

All patients were admitted to hospital via the emergency department, and most were admitted for lung disease management. The average score on the MEWS was 2, indicating that the average patient’s score on admission was not a trigger for urgent medical review. The median score of 7.2 on the DASI is associated with 3.63 METs, indicating that the average patient, at baseline, had low-moderate functional capacity or could walk a block on ground level (2.75 METs) or vacuum (3.5 METs) but could not climb a flight of stairs (5.5 METs) [62].

Most patients had an illness and were taking medications with orthostatic effects. The average patient had morbidity in 7 (range 2 to 12) CIRS-G organ systems with at least one organ system rated at level 4 or extreme severe impairment (*n* = 55; 91.7%). Their mean total scores on the ACCI and the CIRS-G reflect moderate comorbidity [55].

As reported in Table 3, mean scores on the TAP were slightly above the mid-range of the scale (i.e. 2) for both the bed-to-sitting and sitting-to-walking interventions, suggesting that participants perceived both interventions as acceptable. Most patients expressed a preference for the sitting-to-walking intervention.

**Table 3:** Perceived intervention acceptability & preference for 60 study participants

Variable	
TAP score—mean ± SD	
Bed-to-sitting intervention	2.48 ± 0.72
Sitting-to-walking intervention	2.43 ± 0.64
Intervention preference—n (%)	
Sitting-to-walking	26 (43.3)
No preference	25 (41.7)
Bed-to-sitting	9 (15)

*n* number, *TAP* Treatment Acceptability and Preferences, *SD* standard deviation

### Test of statistical assumptions

No major departures from normality or violations of the assumptions underlying the linear and logistic regression analyses were identified. The variance inflation factors (VIF) were all less than 1.3 and the tolerances were all greater than 0.78, indicating that the independent variables were not redundant or correlated. In the linear regression exploring the associations of the patient characteristics with perceived acceptability of the interventions, the assumptions of normal distribution of residuals and homoscedasticity were met, as indicated by visual inspection of residual plots and results of the Kolmogorov-Smirnov and Shapiro-Wilk Tests of Normality.

In the ordinal logistic regressions exploring the associations of the patient characteristics with preference for the interventions, the Chi-squared Goodness-of-Fit test and the Test of Parallel Lines (i.e. testing if the coefficients/slopes are the same across explanatory variable categories) were not statistically significant (*p*-values were greater than or equal to .50) as required for proper model fit.

### Patient characteristics associated with perceived early activity acceptability

Separate linear regressions were applied to explore the associations of patient characteristics (gender, illness severity, comorbidity, illnesses and medications with orthostatic effects, and baseline functional capacity) with perceived acceptability of each of the two low-intensity, early activity interventions (aim 2); these were performed separately for the two comorbidity measures as additional explanatory variables. None of the characteristics were significantly associated (all *P*-values > .05) with perceived acceptability of either intervention.

### Patient characteristics associated with early activity preference

Ordinal regression was applied to explore the associations of the same set of patient characteristics with early activity preference (aim 3); this was performed separately for the two comorbidity measures. Using the ACCI as a measure of comorbidity, illness severity was associated with early activity preference and had a positive coefficient (0.62) with an odds ratio of 1.9 [95% Confidence Interval (CI), 1.04 to 3.30] Wald *χ*^2^(1) = 4.34, *p* = 0.04). In other words, patients with higher illness severity were almost twice as likely to express a preference for the sitting-to-walking intervention compared to the bed-to-sitting intervention or no preference. Medications with orthostatic effects was associated with early activity preference with a positive coefficient (2.29) and an odds ratio of 9.9 [95% CI, 1.30 to 74.8] Wald *χ*^2^(1) = 4.89, *p* = .03. Patients taking medications with orthostatic effects were almost 10 times more likely to express a preference for the sitting-to-walking intervention (while holding constant the model’s other variables). Similar results were found using the CIRS-G as a measure of comorbidity.

## Discussion

Participants’ perceived acceptability of both interventions clustered above the scale midpoint; most preferred the sitting-to-walking intervention (aim 1). While none of the patient characteristics (gender, illness severity, comorbidity, illnesses and medications with orthostatic effects, and baseline functional capacity) were associated with perceived acceptability of the interventions (aim 2), illness severity and medications with orthostatic effects were significantly associated with intervention preference (aim 3). The results are generalizable to cognitively intact older men and women admitted to hospital with an acute medical illness, low-moderate baseline functional capacity and moderate comorbidity.

To our knowledge, this is the first study to identify the early activity intervention preferences of older patients with multiple chronic conditions admitted to hospital with an acute condition. Most (58.3%) expressed a preference for one intervention over the other. The finding concurs with those of meta-analyses indicating that, when offered treatment options, most (≥ 60%) people identify a preference [63, 64]. The few studies that have focused on perceptions of early activity during hospitalization found that some older medical patients have strong beliefs about the need for rest [10, 65], the inappropriateness of activity during illness, and the risks of performing activity in the hospital setting but are motivated to be active by the negative effects of bed rest [11]. The current study adds to this body of knowledge by identifying that, when older medical patients were engaged in a systematic process that involves considering the orthostatic effects of bed rest and critically appraising the acceptability of interventions to address these effects, most (43%) expressed a preference for the sitting-to-walking intervention – only 15% expressed preference for the bed-to-sitting intervention. Clinicians may use this systematic process to help older medical patients make informed decisions around participating in activity during acute illness and hospitalization, and to explore their perceptions of activity. The 8-item TAP scale provides an efficient approach for clinicians to assess older patients’ perceived acceptability of, and preferences for, early activity interventions; the patients’ responses can then be explored in greater depth to clarify any misconceptions about the interventions.

Only 15% of the study participants expressed preference for the bed-to-sitting intervention. Although the ultimate goal is to return patients to their usual level of activity (which in the study sample was walking), the bed-to-sitting intervention provides an alternative to remaining inactive and sedentary for patients who are unable to tolerate walking or refuse to walk during hospitalization. Despite evidence suggesting that the older patient population has varying perceptions of the acceptability of early activity interventions, prior studies have not examined patients’ perceived acceptability of, or preferences for, different early activity interventions. Patients’ inactivity and bed rest in the current acute care environment, where early activity is encouraged, may reflect their personal choices and preferences. Respecting patients’ choices and preferences and offering intervention options aligned with them are key components of patient-centered care [66]. Although the bed-to-sitting intervention requires further testing, its selection by 15% of the study sample suggests that it may be a viable option for sedentary patients. Patients who are provided with interventions that they explicitly expressed a preference for have higher rates of intervention adherence and lower rates of attrition compared to those who are not offered their preferred intervention [67–70].

About 42% of the study participants expressed no preference for the interventions examined in this study. This finding is difficult to interpret but may relate to features of the sample and/or the interventions. Older adults may not have been socialized to actively engage in treatment selection and may have had difficulty expressing their preference for one intervention over another. Also, with the average perceived acceptability score slightly above the mid-range (i.e., between acceptable and very acceptable), some participants did not view the interventions as highly appealing, suggesting the need for additional research to explore, qualitatively, what participants liked or did not like about each intervention.

The current study also adds to the body of knowledge on the patient demographic and health related characteristics associated with perceived acceptability and preference of early activity interventions during acute illness and hospitalization. Participants with higher illness severity were almost twice as likely to express a preference for the sitting-to-walking intervention. Of the two interventions, the sitting-to-walking intervention, which requires patients to get out of bed, can be considered more intensive. Prior research found beliefs surrounding the causes and consequences of a health condition to influence preferences in other patient populations [71]. People with more serious health conditions tend to consider mediation necessary and, consequently, are more likely to select more intensive and aggressive interventions [72, 73]. Accordingly, participants who were sicker on admission may have preferred the more intensive intervention (i.e., sitting-to-walking) because they perceived themselves as sicker and at greater risk of developing orthostatic intolerance and functional decline. They may have viewed the sitting-to-walking intervention as having better potential to prevent these problems.

Clinicians can use the findings to anticipate that older medical patients with higher levels of illness acuity on admission, as measured by the MEWS, will be more likely to prefer the sitting-to-walking intervention, and to not direct patients to the lower-intensity activity intervention. The MEWS is a simple and easy to use tool that uses routinely collected data from which illness acuity can be quickly calculated at the bedside [51] and may be used to anticipate activity preference in clinical practice and future research.

Participants taking medications with orthostatic effects were more likely to express their preference for the sitting-to-walking intervention; however, while significant, the wide 95% confidence interval suggests low precision. Participants taking these medications could be anywhere from 1.3 to 74.8 (95% CI) times more likely to prefer the sitting-to-walking intervention. This imprecision could be related to the low variance on this dichotomous variable; only seven patients (11.7%) were not taking this type of medication and, consequently, the result should be interpreted with caution. Future studies with larger sample sizes would be needed to generate a more precise interval given an expected high prevalence of these types of illness in the target population [39]. Similarly, the sample varied little on illnesses with orthostatic effects, which may account for the lack of statistical significance in this variable.

Gender was not associated with perceived early activity intervention acceptability or preference. This differs from other studies which identified gender-based differences in physical activity preferences, with men being treated for prostate cancer in ambulatory clinics preferring strength- over aerobic-based activity [35]. Although further research is needed, it is possible that the interventions examined in this study were not perceived to differ in terms of their strength and aerobic basis because they were both designed to promote vascular return.

Comorbidity and baseline functional capacity were not associated with perceived early activity intervention acceptability or preference. Although we found no studies examining the relationship that these variables have with perceived intervention acceptability and preference, prior studies have identified patients with higher comorbidity to have lower functional capacity [37]. Our study sample had a moderate level of comorbidity, with extremely severe impairment in at least one CIRS-G organ system, and their low-moderate baseline functional capacity reflects an inability to perform the levels of moderate-to vigorous-intensity physical activity recommended by national activity guidelines for older adults to maintain good health [74]. The interventions examined in this study were perceived as acceptable by this patient population and support future research examining their effectiveness. To the best of our knowledge, only one controlled trial has examined the effectiveness of early activity in the form of a walking program in this patient population [33], supporting the need for further research.

### Strengths and limitations

The results of this study should be interpreted in the context of its aims, which were to explore patients’ perceived acceptability and preference for two early activity interventions prospectively. While a strength of the study was the systematic process used to examine perceived acceptability, the TAP scale does not have validated norms for the target population. Consequently, the subjective responses based on their position along the response options of the TAP scale should be interpreted with caution. Similarly, the sample size was small, and the study should be replicated with a larger sample size.

The study did not measure actual activity performance. Although recent research has found older medical patients’ perceptions and attitudes toward activity during hospitalization to predict their actual activity performance [65], future research is needed to explore patients’ perceived acceptability concurrently as they participate in the interventions and if perceived acceptability predicts actual activity performance during hospitalization.

The interventions have not been tested as stand-alone interventions for their effectiveness in preventing orthostatic intolerance, bed rest dependency or functional decline in the study’s target population. Further research to test their effectiveness should be conducted prior to adopting the interventions in practice. Future research should also examine the relationship between older hospitalized patients’ attitudes toward activity and their acceptability of the early activity interventions to further understanding of factors that influence acceptability and to identify patient characteristics on which to tailor early activity.

## Conclusions

Assessing the perceived acceptability and preferences are increasingly acknowledged as essential in the design, evaluation, and, ultimately, implementation of healthcare interventions but prior studies did not examine older hospitalized patients’ perceived acceptability of, and preferences for, early activity interventions and they demonstrated poor feasibility. The two low-intensity early activity interventions that we designed and examined in this study were perceived as acceptable to older patients admitted to hospital with an acute medical illness, low-moderate baseline functional capacity and moderate comorbidity. Patient characteristics were not significantly associated with perceived intervention acceptability, but illness severity was significantly associated with intervention preference. The results support future research examining the effectiveness of the interventions in older hospitalized medical patients admitted to hospital with an acute medical illness, low-moderate baseline functional capacity and moderate comorbidity.

## Abbreviations

ACCI: Age Adjusted Charlson Comorbidity Index
ACE: Acute Care for Elders
CAM: Confusion Assessment Method
CI: Confidence Interval
CIRS-G: Cumulative Illness Rating Scale – for Geriatrics
DASI: Duke Activity Status Index
MET: Metabolic equivalent
MEWS: Modified Early Warning Score
MMSE: Mini Mental Status Exam
n: Number
OR: Odds ratio
*p*: Probability
SD: Standard deviation
TAP: Treatment Acceptability and Preferences
VIF: Variance inflation factors
*χ*^2^: Chi-square
